# The Deployment and Utilization of the Allied Health Professions Support Workforce: A Scoping Review

**DOI:** 10.2147/JMDH.S460543

**Published:** 2024-05-13

**Authors:** Sarah Etty, Beverly Snaith, Daniella Hinchcliffe, Julie Nightingale

**Affiliations:** 1 Department of Allied Health Professions, Sheffield Hallam University, Sheffield, UK; 2 Faculty of Health Studies, University of Bradford, Bradford, UK; 3 Department of Radiology, Mid Yorkshire Teaching NHS Trust, Wakefield, UK

**Keywords:** allied health personnel, workforce, scope of practice, assistant, support worker

## Abstract

The demand for healthcare services internationally continues to increase, exacerbated by patient backlogs resulting from the COVID-19 pandemic and the difficulties in recruiting and retaining healthcare staff. These difficulties have led to increased interest in workforce redesign, and the upskilling of existing staff in all areas of healthcare, including within the Allied Health Professions (AHP). Clinical support staff are a key component of workforce redesign, yet little has been documented on the utilization of this workforce across the wide range of professions that collectively form the AHP workforce. Existing research is also unclear due to the variety of titles used to describe them (eg, allied health assistants, therapy assistants, etc). This study aimed to review how Support Workers and Assistant Practitioners (SWAPs) are utilized within the AHP professions. Electronic databases (MEDLINE, CINAHL complete, Scopus, and Google Scholar) were searched to find English Language primary research articles that explored the deployment of clinical support staff within Allied Health. Following the scoping review methodology, data from each study were analyzed in terms of design, key findings, and implications. A quality assessment was also completed. Thirty-nine articles met the eligibility criteria. Studies were undertaken in Australia, UK, and USA, and covered a range of AHPs and methodological approaches. Most articles employed qualitative methods, with highly variable research quality identified. Key findings were that cost-effectiveness of this workforce has not been formally evaluated in any setting or AHP discipline, and that support workers are a largely underutilized staff group potentially due to inconsistencies in their deployment and scope of practice, and the lack of a clear career pathway. Rigorous, quantitative, and mixed methods research into the deployment and impact of this staff group is needed in order to gain a clearer understanding of how they are optimally utilized across the different AHP disciplines.

## Introduction

The pressure and demand on healthcare services internationally continues to rise, exacerbated by the impact of COVID-19. The World Health Organization highlighted that nearly all countries reported significant disruption to essential health services as a result of the pandemic,^1^ and many countries across Europe reported substantial backlogs even when care had been restored to pre-pandemic levels.^2^ Prior to the pandemic, some countries were already facing significant clinical pressures and ever-increasing waiting lists due in part to difficulties in the recruitment and retention of registered staff.^3^ The COVID-19 pandemic worsened these staff shortages due to either infection or self-isolation requirements,^4^ and the necessary cancellation of non-urgent care further added to already extensive waiting lists.^4^,^5^ These pressures mean that activity focused on workforce redesign solutions is ever more crucial. Upskilling (expanding an existing skillset through training and education) has become more widespread, with Support Workers and Assistant Practitioners (SWAPs) taking on some tasks originally undertaken by registered staff. This has resulted in greater utilization of SWAPs in some countries, settings, and professions, yet there is uncertainty around how best to deploy this workforce, underpinned by international variations in their skills and knowledge.

Allied Health Professions (AHPs) in most countries comprise the third largest healthcare workforce group behind medicine and nursing.^6^ The professions included in the AHP definition vary internationally and, in some cases, even between individual regions. In the United Kingdom (UK), for example, AHPs in England comprise 14 professionally autonomous professional groups, educated to bachelor’s degree level or equivalent, that

Provide system-wide care: they are involved in assessing, treating, diagnosing and discharging patients across health, social care, housing, education, early years, schools, the criminal justice system, independent and voluntary sectors.^6^

In Wales, radiographers and operating department practitioners (ODPs) are excluded as they are considered healthcare scientists, whilst Scotland and Northern Ireland include radiographers as AHPs but not ODPs. Ireland only defines eight AHPs. Conversely, Canada, the United States of America (USA), and Australia include a wider range of AHPs, including psychologists, counsellors, and psychotherapists in the latter two countries.^7^,^8^ Despite the variation, most definitions concur that AHP professions are “distinct from medicine and nursing”;^8^ however, they experience similar workforce pressures to those affecting the larger healthcare professional groups, and delegation of responsibilities is already occurring in a range of AHP settings.

The unregistered AHP support workforce includes a range of job titles that differ depending on the profession and the country. For example, in Australia and USA, the broad Allied Health Assistant (AHA) title is often used, whereas in the UK, such staff can be referred to by a range of different names such as clinical support workers, health care assistants, or medical assistants.^9^ Within individual AHP professions, such as physiotherapy, there are a very wide range of job titles. Within the UK these include titles such as physiotherapy assistant, rehabilitation assistant, technical instructor, or physiotherapy technician.^10^ The wide variation in job titles also suggests that role content varies widely from administration to clinical tasks. Further variation in job titles occurs at entry, senior and advanced levels of practice (Table 1). Given the high level of variation in support workforce titles, this article will refer to this workforce using the UK specific terms of Support Workers and Assistant Practitioners (SWAPs).

**Table 1:** Comparison of AHP Support Workforce Role Titles Across Three Different Countries

	Australia^11^	UK^12^	USA^13^*
**Generic Name**	Allied Health Assistant (AHA)	Clinical Support Worker	Allied Health Assistant
**Entry level**	Level 1 AHA	Support Worker	Basic Healthcare Worker
**Senior**	Level 2 AHA	Senior Support Worker	Allied Health Assistant
**Advanced**	Level 3 AHA	Assistant Practitioner	Advanced Allied Health Assistant

Note: *USA allied health assistant roles vary between states.

A number of literature reviews have previously been published on the SWAP workforce. A systematic review^14^ published in 2010 assessed the roles and responsibilities of SWAPs from several AHP professions in Australia, UK, and USA, concluding that variability in their deployment was influenced by work setting, profession, and relationship between therapist and assistant. Another systematic review^15^ published in 2013 explored the implementation of advanced SWAPs and found that only 4 of the 53 studies reviewed investigated the effectiveness of these roles. A more recent systematic review^16^ published in 2021 investigated the clinical and cost-effectiveness of delegation by AHPs to SWAPs and included the views of both healthcare professionals and patients. This review highlighted the lack of studies addressing the cost-effectiveness of delegation, and the importance of clear training pathways in successful delegation. None of the previous reviews have considered the ways in which SWAPs are deployed and there is a lack of clarity in what constitutes effective deployment. Therefore, the aim of this contemporary review is to explore how SWAPs are utilized by a range of AHPs across different international contexts, mapping out key similarities and differences between the different AHPs in their models of deployment.

## Method

This study addressed an exploratory research question, and therefore the most appropriate methodology was a scoping review. Scoping reviews differ from systematic reviews in that they cover much wider topics and explore the existing literature by summarizing and synthesizing data to gain a general understanding of a research area.^17^ This review was guided by the recommendations of Mak & Thomas^18^ in their published guidance.

### Search Strategy

The search strategy for this review was developed with the assistance of an experienced information specialist. A literature search was performed using MEDLINE, CINAHL Complete, and Scopus to identify any empirical peer-reviewed articles regarding the deployment of the support workforce in Allied Health Professions. The PEO (population, exposure, outcome) framework was employed to structure the search terms (Table 2) which were combined using Boolean operators (eg, “and” “or”). Full search strings for database searches can be seen in Appendix 1. Literature searches were also repeated using Google Scholar to widen the search, and due to the volume of results generated in Google Scholar only the first 100 results for each search (when sorted in order of relevance) were progressed to the screening stage.

**Table 2:** PEO Framework

PEO Framework	Search Terms
Population: Allied Health Professions	Allied health, (all UK allied health professions – see Appendix 1 for full search strings)
Exposure: Support workers and Assistant Practitioners	Assistant, support
Outcome: Impact, deployment, role	Professional role

**Figure 1:**
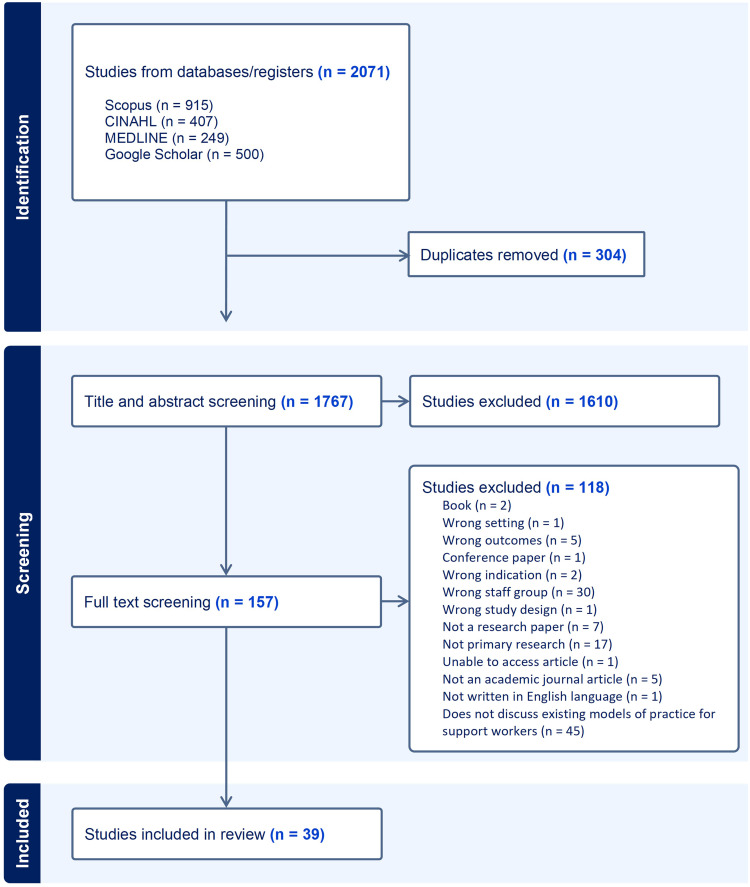
PRISMA flow chart showing screening and selection process for SWAP deployment.

**Notes**: Adapted from Page MJ, McKenzie JE, Bossuyt PM, et al. The PRISMA 2020 statement: An updated guideline for reporting systematic reviews. *Int J Surg*. 2021;88:105906.^19^

### Eligibility Criteria

Only peer-reviewed, academic journal articles written in the English language were eligible for inclusion. Further eligibility criteria included articles reporting primary research findings and discussing the roles and utilization of SWAPs working in AHP settings, ie, any setting in which allied health professionals deliver care, including hospital and community settings. Date was not used as a limiter within the literature search.

### Study Selection

The screening process (title and abstract screening, followed by a full text screening) was completed using Covidence,^20^ a web-based literature screening and data extraction tool. Two members of the research team independently completed each stage of the screening, engaging in discussion to reach consensus on each article in the case of conflicting decisions. For the Google Scholar search results, the title and abstract screening was completed by one member of the research team, with subsequent screening steps consistent with the previous database search.

### Data Extraction and Quality Assessment

An assessment of article quality was not identified in the guidance for completing scoping reviews published by Mak & Thomas;^18^ however, it was included as the full text screening phase highlighted significant variation in quality. The assessment was completed using the Quality Assessment with Diverse Studies (QuADS) appraisal tool,^21^ which applies to both quantitative and qualitative studies. Data extraction and quality assessment were completed by one reviewer and checked for accuracy by a second reviewer using Covidence.^20^ The data were then subsequently downloaded with the extraction covering all areas relevant to the research aims.

## Results

### Study Characteristics

A total of 2071 documents were retrieved for review. Following the exclusion of duplicate records and screening, 39 peer reviewed articles were considered appropriate for data extraction (Figure 1). The year of publication ranged from 1998 to 2023. Included studies originated from either Australia, Canada, USA, or Europe, and encompassed a range of different AHPs (Table 3). Study participants also varied and included managers, AHPs, SWAPs, and allied health care consumers.

In Australia, some SWAPs work alongside a defined profession, with varying levels of responsibility ranging from a largely administrative role^22^ to making clinical decisions and monitoring patients alongside their registered counterparts.^23^ Many were employed in broad roles covering care and therapist-specific functions but aligned with many different registered professionals. This was similar for rehabilitation assistants in the UK^24^ who mostly work alongside physiotherapy and occupational therapy colleagues. However, this is not the case for Assistant Practitioners (advanced-level support workers) in the UK who are more commonly aligned to a single allied health discipline.^25–33^

**Table 3:** Extraction Table for Selected Articles

Citation	Country	Method Description	Profession/Service	Sample Size	Participants	Sector	Setting	QuADS Score / 39
Armstrong et al (2012)^34^	USA	Electronic survey	Speech and language therapy	415	Other: Speech-language pathologists (SLPs), Speech-language pathologist assistants (SPLAs)	Schools	Mixed	25
Bennion & Irvine (2011)^35^	UK	Qualitative, descriptive and exploratory. Focus group and follow up interviews. Framework analysis for data analysis.	Radiography	10	Radiology managers	Hospital	Not stated	27
Cartwright et al (2021)^22^	Australia	Prospective comparison of multiple workforce models, including usual practice, to measure the efficiency of each.	Radiography	6	Support Workers; Administrative assistant; radiographers.	Hospital	Urban	25
Eliassen & Moholt (2023)^36^	Norway	Observations and follow up interviews	Physiotherapy	14: 7 PTs, 7 HTs	Other: Physiotherapists (PTs) and Home Trainers (HTs)	Mixed	Not stated	25
Eliassen et al (2020)^37^	Norway	Observation and interviews (qualitative)	Physiotherapy	21: 7 users, 7 PTs, 7 HTs	Other: Home trainers (HTs) and Physiotherapists (PTs)	Mixed	Not stated	33
Ellis & Connell (2001)^38^	UK	Semi-structured interviews	Physiotherapy	36 (18 assistants and 18 physiotherapists)	Other: Physiotherapy assistants and their supervising physiotherapists	Mixed	Mixed	27
Ford (2004)^39^	UK	Questionnaires and follow up focus group	Radiography	16 for surveys, 7 for focus group	Radiology Service Managers	Hospital	Not stated	26
Frowen et al (2021)^40^	Australia	Pre and post-test study	Speech and language therapy	61	Other: Head and neck cancer patients undergoing radiotherapy	Hospital	Not stated	31
Huglin et al (2021)^41^	Australia	Literature scan, scoping survey of key stakeholders, and individual and focus group semi structured interviews. Thematic analysis was used for interview data.	Multiple AHPs	119	Allied Health Assistants; AHPs; Allied Health Leaders; AHA certificate training program educators and managers; AHA certificate training program students; and consumers of Victorian, health, disability and aged care services.	Mixed	Mixed	34
King et al (2022)^42^	Australia	Semi-structured interviews	Multiple AHPs	21	Allied Health Assistants	Hospital	Mixed	29
Kiss et al (2019)^43^	Australia	Pre and post-test study	Nutrition/dietetics within a cancer treatment clinic	91	Other: Head and neck cancer patients	Hospital	Not stated	18
Knight et al (2004)^24^	UK	Case study approach of 13 rehabilitation assistants using time sheets, observation, the think-aloud technique, and semi-structured interviews	Multiple AHPs	13	Rehabilitation Assistants, and team leaders	Mixed	Not stated	23
Kuipers et al (2015)^44^	Australia	Role audit	Multiple AHPs	41	Allied Health Assistants; AHPs; line managers/team leaders; health care team members	Mixed	Mixed	10
Le Cornu et al (2010)^45^	UK	Mixed methods - questionnaire and semi-structured telephone interviews	Dietetics	18 DSWs, 62 dietitians for survey, sub-sample of 6 DSWs and 6 dietitians for interviews	Support Workers; Dietitians	Mixed	Not stated	26
Leach & Wilton (2009)^25^	UK	Semi-structured questionnaires and interviews	Multiple AHPs; Nursing	13	Assistant Practitioners and Managers	Mixed	Not stated	8
Lin et al (2007)^46^	Australia	Cross-sectional survey of AHP managers, TAs, TA coordinators and AHPs administered via either telephone interview or email.	Multiple AHPs	14	Allied Health Assistants	Mixed	Rural/remote	21
Mackey & Nancarrow (2005)^26^	UK	Focus group semi-structured interviews	Occupational Therapy	17	Assistant Practitioners; Supervisors, Team managers, service users	Community	Not stated	19
Mackey (2004)^47^	UK	Focus group interviews	Occupational Therapy	36	Qualified and Unqualified Occupational Therapy Practitioners	Community	Not stated	22
McCartney et al (2005)53	UK	Case studies - questionnaires and interviews	Speech and language therapy	5	Other: Speech and language therapists	Schools	Urban	28
Mickan et al (2018)^48^	Australia	Exploratory semi-structured interviews	Multiple AHPs	12	Senior Allied Health clinicians and academics	Mixed	Not stated	25
Nancarrow et al (2015)^49^	Australia	Semi structured interviews, focus groups, documentary analysis, workload audit	Speech and language pathology	13	Speech language pathology assistants, Speech and language pathologists, service managers, service users and their carers.	Hospital	Not stated	19
Palmer et al (2018)^27^	UK	Electronic surveys	Radiography	108	Assistant Practitioners	Hospital	Not stated	29
Pearce & Pagett (2015)^50^	Australia	Literature review, informal telephone and Email consultation, focus groups, online survey, semi structured interviews	Multiple AHPs	36 AHAs, 23 AHA managers	Allied Health Assistants; AHAs; assistants’ managers.	Mixed	Not stated	16
Penner et al (2020)^51^	Canada	Quantitative surveys and qualitative focus groups	Physiotherapy; Occupational Therapy	89 for surveys (56 therapists, 37 assistants), 30 for focus groups (17 therapists, 13 assistants)	Other: OTs and Physiotherapists, Occupational therapist assistants and Physiotherapist assistants	Hospital	Urban	28
Pinson et al (2023)^52^	Australia	Time motion survey and qualitative interviews	Radiography	4	Medical Imaging Assistants	Hospital	Mixed	30
Price & Le Masurier (2007)^28^	UK	Quantitative, structured questionnaire	Radiography	177	Radiology managers	Hospital	Not stated	21
Price et al (2015)^29^	UK	Telephone interviews	Radiography	20	Imaging department managers	Hospital	Not stated	18
Rushton et al (2022)^23^	Australia	Semi-structured interviews	Dietetics	23	Dietitian assistants; dietitians; management staff; food services staff.	Hospital	Mixed	27
Russell & Kanny (1998)^53^	USA	Questionnaire (both quantitative and qualitative questions)	Occupational Therapy	366	Occupational Therapy Assistants and Occupational Therapists	Mixed	Not stated	29
Snaith et al (2018)^30^	UK	Electronic surveys	Radiography	193	Assistant Practitioners	Hospital	Mixed	24
Snowdon et al (2022)^54^	Australia	Time-motion study	Multiple AHPs	51	Allied Health Assistants	Mixed	Not stated	26
Somerville et al (2015)^55^	Australia	Mixed methods - focus groups and quantitative survey	Multiple AHPs	2449	Allied Health Assistants; AHPs.	Mixed	Mixed	16
Somerville et al (2018)^56^	Australia	Mixed methods - focus groups and quantitative survey	Multiple AHPs	1247	Allied Health Assistants; AHPs	Mixed	Not stated	18
Stewart-Lord et al (2011)^31^	UK	Scoping exercise, survey	Radiography; Radiotherapy	167	Assistant Practitioners	Hospital	Not stated	22
Stewart-Lord et al (2014)^32^	UK	Semi-structured interviews	Radiography; Radiotherapy	38	Assistant Practitioners	Hospital	Not stated	27
Stute et al (2014)^57^	Australia	Pilot study and role audit	Multiple AHPs	41	Allied Health Assistants	Mixed	Mixed	25
Wilberforce et al (2016)^58^	UK	Pilot of time use (diary tool evaluation)	Occupational Therapy	151	Other: Occupational therapists and assistants	Mixed	Urban	31
Wood et al (2011)^59^	Australia	Semi structured interviews	Multiple AHPs; Community Rehabilitation	31	ACRAs; health professionals; rehabilitation clients.	Community	Not stated	20
Zelenyanszki et al (2022)^33^	UK	Service evaluation	Radiography	8	Assistant Practitioners; Registered mammographers; programme manager; office manager.	Mobile breast screening units	Not stated	25

### Quality of Research

The quality scores are reported in Table 3. There is no recommended cut-off score for low- or high-quality studies for the QuADS appraisal tool,^21^ as the intended purpose is to generate discussion of the different measures of quality.^21^ The majority of articles included in this review utilized a qualitative research design. QuADS scores were generated by calculating the sum of scores for all 13 items, with each individual item able to score between 0 and 3, with an overall maximum QuADS score of 39. The QuADS scores varied widely, ranging from 8/39, to 34/39, with a mean score for all articles of 23.9 (Table 3). Clear descriptions were provided of the research setting and population, with articles scoring a mean of 2.77 (out of 3) for this criterion, and study designs were appropriate to address the research aims (item mean score = 2.72). However, only 6 studies provided adequate justification for the analytic method used (item mean score = 0.44), and 46% of studies did not show any evidence of consideration of stakeholders (item mean score = 0.67). As this review aimed to scope all existing primary research on the deployment of SWAPs in AHP settings, the quality of literature was not used as an exclusion criterion.

### Deployment

The areas of deployment varied widely for SWAP roles. Eight articles showed that some SWAP roles were primarily patient-facing,^24^,^46^,^49^,^53–55^,^59^ with two of those eight articles showing additional clerical or administrative tasks as a part of their workload.^53^,^55^ One paper^42^ reported that many SWAPs viewed the patient-facing, therapeutic aspect of their role as more important than the administrative function. One article^51^ described SWAPs feeling excluded from key discussions and decisions, which one registered practitioner attributed to SWAPs being “workhorses”, and pulling them from their duties to attend meetings has a big impact on service delivery.

Deployment varied in the setting (rural versus metropolitan),^44^,^55^ allied health discipline,^55^ and team structure.^36^,^37^ In rural settings, the proportions of SWAPs were reported to be higher than in metropolitan settings,^55^ and they performed proportionally more non-clinical tasks.^44^ Two articles, which appear to report on the same study, identified that teams either followed a hierarchical, fixed structure, or a symbiotic, flexible structure.^36^,^37^ SWAPs in hierarchical teams followed the instructions of registered practitioners, often worked independently on “less important” tasks, were limited in their scope of practice, and were presented with fewer learning opportunities. Symbiotic teams allowed SWAPs to contribute more, take more of a lead, and therefore build competence in decision-making. Conversely, two separate articles on SWAPs in different allied health disciplines^40^,^43^ reported similar deployment, providing screening and basic education for non-complex patients, and referring complex patients to registered practitioners. However, these two differing disciplines were both operating in a head and neck cancer clinic.

Four articles reported flexibility within the SWAP workforce, including both specialism and role; recognizing that staff are sensitive and adaptable to the needs of the department.^27^,^30^,^38^,^52^ For example, SWAPs were assigned to cover physiotherapy work on wards when the department was short staffed, however, the tasks were then relinquished once the physiotherapist returned.^38^ However, even within a single profession there was significant variability in deployment, for example within radiography, where SWAP roles were either utilized widely in a rotational capacity across numerous settings^52^ or restricted to individual imaging modalities such as computed tomography or ultrasound.^31^ In pediatric speech and language pathology, SWAPs can work across varying numbers of schools, with the majority working between 2 schools, compared with 12% working across 5 or more.^34^

### Scope of Practice

The articles in this review demonstrate that SWAPs work across a variety of different AHPs, including radiography, physiotherapy, occupational therapy, dietetics, and speech and language therapy. Two articles highlighted that SWAPs in Australia also work in Exercise Physiology, Podiatry, Prosthetics/orthotics, Psychology, Social Work, Pastoral Care, Orthoptics, Neuropsychology, Music Therapy, and Audiology.^55^,^56^ Six articles reported SWAPs working across more than one discipline, as a transdisciplinary assistant,^24^,^42^,^46^,^51^,^54^,^59^ with physiotherapy and occupational therapy as the most common combination. Three Australian articles described different levels of the SWAP roles,^23^,^50^,^57^ with two of those three also describing the scope of practice associated with each,^23^,^50^ with one article defining the levels as being trainee, full scope, and advanced scope.^57^ There was some contradiction in the scope of practice, with four studies describing a broader scope.^24^,^49^,^54^,^55^ In some cases, this included providing training and education for a range of colleagues^42^ and contributing their ideas to the improvement of the department.^25^ However, 11 articles highlighted restrictions placed on this workforce group,^22^,^23^,^26–29^,^32^,^43^,^48^,^53^ including keeping complex patients under the care of registered practitioners,^23^ and working under their supervision.^29^,^32^,^35^ Under-utilization of this workforce was also demonstrated in 11 of the studies.^24^,^31^,^35^,^44^,^45^,^47^,^48^,^52^,^55–57^ For example, one audit^44^ found that only around 10% of SWAPs were performing all the duties expected of them, and a qualitative study found that some SWAPs were not being allowed to utilize the learning and skills that they had acquired.^48^ In contrast, five articles reported that the SWAP workforce had a level of personal autonomy,^24^,^25^,^42^,^52^,^59^ and a further article^60^ demonstrated that speech and language pathology SWAPs carry their own caseloads, are encouraged to be autonomous by registered practitioners, and deliver therapy alone following assessment by a registered practitioner. This study also showed that after some time had passed, their scope of practice was broadened to include the writing up of reports to be checked by registered practitioners. Another article^34^ on SWAPs in speech and language pathology showed that SWAPs had a broad scope of practice, with the majority of their time spent on direct services to students.

Scope of practice was, however, found to vary depending on certain factors, for example on the practice of the supervising registered practitioner, the needs of the individual service^38^ and the type of team structure.^36^ However, in one article, differences in scope of practice between SWAPs working in the same service and setting were not explained.^60^

Managers in one study agreed that the scope of practice for SWAPs could be extended, but that restrictions should be in place.^39^ Another article evaluated the SWAP role as an extension of the AHP, rather than a replacement for them.^24^ Again, the scope of practice was cited as being variable depending on sites or organisations,^27^,^30^,^38^,^59^ and a lack of clarity was reported around the roles and boundaries^24^,^41^,^42^,^48^,^57^ of those in SWAP roles. Additionally, the findings of seven studies indicated that SWAPs are working beyond their scope of practice.^27^,^30–32^,^50^,^53^,^57^ In contrast, two articles highlighted cases where role boundaries are clearly defined,^49^,^57^ and two further articlesreported role boundaries to be well understood by those working in the roles.^23^,^45^ Interestingly, one article found that National Vocational Qualifications (NVQs), a work-based qualification in the UK, were seen as useful in guiding the scope of practice boundaries,^47^ whereas another found that there was poor understanding and undervaluing of SWAP qualifications.^26^

### Role Differentiation

Three articles examined the activities undertaken by SWAPs and the registered practitioners they work alongside,^34^,^38^,^58^ which enabled comparisons to be made. One article^34^ found that in speech and language pathology, caseloads are larger for SWAPs than for registered practitioners, and that SWAPs spend much less time on more complex activities such as evaluations, dynamic assessments, and little to no time conducting any supervisory activity or making referrals. However, despite these differences, there was not a great deal of variation in the many other activities completed, and the time spent on them. An article on SWAPs in occupational therapy^58^ found that SWAPs spent significantly more time delivering therapy and completing administrative tasks than registered practitioners, and significantly less time on multidisciplinary working; however, time spent undertaking direct care did not differ significantly between the two groups. In physiotherapy, one article^38^ showed that the difference between SWAPs and junior physiotherapists is assessment skills and responsibility for patients, yet this changed depending on setting; in elderly care and rehabilitation, supervisors felt that assistants were undertaking more of the role of junior physiotherapist, whereas in outpatients, SWAPs were taking on new extended roles, and in community, they were extending the availability of physiotherapy care. An article^51^ examining the role of SWAPs working across both physiotherapy and occupational therapy found that SWAPs acted as the link between the two professions.

### Differences in Setting and Discipline

Five studies demonstrated variations in deployment differing by geographical or healthcare setting. For example, one article^44^ showed that SWAPs working in rural and regional positions in Australia had significantly less access to in-service training, had fewer formal training plans in place, and were not working to the full scope of their positions. While other differences were not statistically significant, rural/regional SWAPs tended to perform proportionally more non-clinical tasks and were provided with less supervision. Two linked articles examined the deployment of SWAPs in Victoria, Australia. The first,^55^ published in 2015, explored deployment in rural, remote, and metropolitan services, whereas the second,^56^ published in 2018, examined this in community and ambulatory healthcare services. The amount of time spent by registered practitioners completing tasks that could be delegated to SWAPs increased from 17% in the earlier study, to 24% in the later study. The later study also showed lower proportions of SWAPs as a part of the allied health workforce in community and ambulatory settings. The earlier study also demonstrated that the proportions of SWAPs within the wider workforce varied depending on geographical location, in that rural and remote locations tended to have higher proportions than metropolitan locations. Another study^38^ found that the healthcare setting determined whether SWAPs were allocated their own caseload; the majority were allocated a specific caseload, but SWAPs working in outpatient settings were not. The reason for this was cited as the brevity of treatment in outpatient settings. A study investigating SWAP roles within physiotherapy and occupational therapy^51^ showed that management structure differed between community and hospital settings. In the community, SWAPs are under the supervision of the therapist, but not in the hospital, and it was noted that this can make providing feedback to SWAPs difficult for therapists.

Variations in the level of delegation by different AHP professions were highlighted, with registered practitioners in exercise physiology and dietetics being more likely to delegate patient-related tasks than those in physiotherapy, psychology, and podiatry.^54^ The same study also found that tasks in acute hospital settings were more likely to be delegated than those in community settings,^54^ perhaps influenced by the potential to escalate to more senior colleagues. Additionally, another study^56^ showed that SWAPs were most underutilized in podiatry and utilized more effectively in audiology. Five articles reported supervision to be variable across numerous disciplines,^27^,^31^,^46^,^53^,^57^ and a further reported that team structure was also variable.^42^

### Factors Influencing Effective Deployment

Table 4 shows factors influencing the effective deployment of SWAPs by country. One article^41^ suggested that role definition, defined scope of practice, and effective delegation are factors that contribute to the optimal utilization of the SWAP workforce. Key drivers for successful delegation and deployment were identified to be effective communication,^23^,^59^ sufficient training, appropriate clinical governance,^23^ and effective supervision.^49^ One article^23^ reported that challenging workloads and increasing demands are both barriers to, and enablers of, delegation. Reluctance to delegate tasks that were traditionally completed by registered practitioners was noted as an impediment to the full utilization of support and assistant staff in five separate articles.^23^,^42^,^48^,^56^,^57^ Trust in SWAPs’ competence enabled successful delegation,^23^ yet it was also found that SWAPs felt that they had to earn this trust.^42^ The likelihood of registered practitioners to delegate tasks to SWAPs was found to differ between the different allied health disciplines.^54^ Time and experience lead to increased use of SWAPs^59^ and successful extension of the scope of practice.^33^ Uncertainty may be an element that contributes to registered practitioners’ reluctance to delegate, as lack of clarity around role boundaries was reported,^26^,^47^ alongside ambiguity around the verification of SWAPs’ competence, and poor understanding of their qualifications.^26^ One article,^24^ however, described multidisciplinary SWAPs as demonstrating high-level reasoning and a thorough understanding of the rehabilitation process.

**Table 4:** Factors Influencing Effective Deployment and Barriers to Career Progression by Country

	Factors Influencing Effective Deployment	Career Pathway Barriers
**Australia**	Role definition Defined scope of practice Effective delegation Effective communication Sufficient training Appropriate clinical governance Effective supervision Challenging workloads Increasing demand Trust in SWAPs’ competence SWAPs having to earn trust of registered practitioners Time and experience Perspectives of registered practitioners (towards delegation) Positive relationships between registered practitioners and SWAPs	Lack of a defined career pathway to qualified roles Limited opportunities to gain experience Lack of a formal training plan Insufficient time for training Limited access to and support for training
**USA**	Mixed attitudes towards SWAPs	
**Canada**	SWAPs having a multidisciplinary role Tasks delegated from external professions	Limited access to and support for training SWAP part-time status
**UK**	Time and experience Lack of clarity around role boundaries Ambiguity around SWAP competence Poor understanding of SWAP qualifications Perspectives of registered practitioners (scope creep) Registered practitioners feeling unprepared to supervise SWAPs Loss of job satisfaction for registered practitioners Perspective of SWAPs being a “cheap replacement” SWAPs feeling competent and involved in decision making	Absence of a career framework Limited opportunities for progression Lack of recognized qualifications available for their role
**Norway**		Limited access to and support for training

The perspectives of the different staff groups may also affect the successful utilization of SWAPs; mixed attitudes towards this staff group were reported,^53^ with one article suggesting that managers and assistants are more likely to have a positive attitude towards delegation than registered practitioners.^23^ Other perspectives from registered practitioners included feeling threatened by role extension of SWAPs, the so-called scope creep,^26^,^47^ and being unprepared for the expectation for them to provide supervision for this new staff group.^29^ Managers also perceived registered practitioners to experience a loss of job satisfaction when delegating patient-facing work they had previously been responsible for.^26^ Additionally, one article^32^ found that SWAPs can be considered a cheap replacement for registered practitioners, which can cause frustration for both groups. Further, one study^51^ exploring SWAPs working across both physiotherapy and occupational therapy reported that the dual role of SWAPs can make role boundaries unclear, cause conflicting loyalties, and create unrealistic competency expectations. It also described difficulties arising as a result of nursing staff also delegating tasks to the SWAPs, as this was not part of their role. However, positive relationships between radiographers and SWAPs were reported,^52^ and additionally, most SWAPs reported feeling competent and involved in decision-making.^31^

### Career Pathway

Entry routes into support and assistant roles were varied, with one article reporting use of a “grow your own” approach,^31^ meaning that staff are hired at entry-level posts and supported to train and develop to progress into more skilled roles. Other pathways into SWAP roles were reported, the most common of which in the UK was the foundation degree for radiography assistant practitioners,^30^ and the NVQ (National Vocational Qualification) for radiography support workers.^39^ A USA speech and language study^34^ found that 87% of SWAPs had a bachelor’s degree, and 14% had a master’s degree. In one diagnostic radiography study, the majority of managers felt that SWAPs should be able to progress their career to qualify as a registered professional, but with sufficient time delay.^39^ Other research^45^ found that dietetic SWAPs and dietitians can envision the dietetic SWAP role developing further. However, nine articles identified barriers to career progression for the SWAP workforce; the absence of a career framework;^30^ the lack of a defined career pathway to qualified practitioner;^50^ limited opportunities for progression^27^,^32^ and gaining experience;^57^ having no formal training plan;^57^ limited access to and support for training,^36^,^51^ having to leave employment to gain qualifications;^30^,^35^ insufficient time for training;^57^ part-time status;^51^ and a lack of recognized qualifications available for their role.^45^ Career pathway barriers by country can be seen in Table 4. One article^47^ identified that a clear career pathway is essential for the retention of the SWAP workforce and that this workforce demonstrates a desire to develop in their roles.^52^ Furthermore, another study showed that registered practitioners were significantly more satisfied than SWAPs with many career development aspects of their role.^41^ Only one study^46^ identified a specified training route (for therapy SWAPs) that had been developed following their research project, though a further study proposed a career pathway with elements aimed to optimize the utilization of SWAPs,^41^ which included three grades of the role, and multiple options for further progression such as into the registered allied health professions or leadership roles. Another^38^ showed that SWAPs can be graded at a higher level when taking on more independence, with SWAPs adopting a greater role in the management of both patients and a ward. This article also identified evidence of extended practice in community and pediatric settings, with SWAPs managing their own individual caseload with minimal supervision.

### Impact on Service

While none of the articles directly measured cost-effectiveness, some were able to demonstrate benefits to service effectiveness resulting from the use of SWAPs. These included a shorter length of stay for patients,^22^,^35^ reduced costs due to SWAPs being cheaper to employ,^22^,^35^ increased service capacity,^37^,^60^ and the release of registered practitioners through role substitution.^22^,^33^,^35^,^49^ Additionally, a clinically and statistically significant improvement in all dimensions of patient satisfaction was observed following the implementation of a nutritional SWAP role.^43^ There was general agreement among some staff that this workforce contributes to a more cost-effective way of working,^25^,^45^ whereas others showed more guarded perspectives.^29^,^53^ Although one study demonstrated that registered practitioners perceived the use of SWAPs to be beneficial, potential challenges to this were the difficulty for registered practitioners in planning and monitoring therapy, and the changes to registered practitioners’ work patterns.^60^ However, benefits to patient care^25^,^51^ and improved client satisfaction and client outcomes^59^ were noted as an important impact of the use of SWAPs. Following the implementation of a speech and language pathology assistant role, one article commented^40^ that there was an increase in patient satisfaction post-implementation (although this did not reach statistical significance), and improved patient perceived benefit, interpersonal skills, and overall satisfaction. The same study also found that the introduction of this role increased registered practitioners’ time spent with complex, high risk, post-op patients, and increased the number of less complex patients seen by either registered practitioners or SWAPs.

## Discussion

This scoping review explored how SWAP roles are utilized by a range of AHPs, working in varying settings within different international contexts, highlighting key similarities and differences between the different AHPs in their models of deployment. The number of articles on this topic was relatively limited with many of those scrutinized at the full text stage not describing this workforce, its deployment or reflecting previous review papers. One of the primary findings of this review is that there is considerable inconsistency in the role titles, scope of practice, and deployment of SWAPs internationally. The majority of included articles were UK and Australian studies which may suggest an increased use of SWAPs in these countries, or it may simply reflect the exclusion of any articles not written in the English language in the literature search. However, an alternative explanation suggests that SWAPs are a relatively new addition to the international allied health workforce, and the UK and Australia have either progressed further in their implementation of the SWAP workforce than other countries, or are more mature in their research on this topic, with multiple recurring authors noted.

### Quality of Research

Research was mostly qualitative, with interviews being the most common data collection method. Some of the articles, however, employed quantitative methods such as time-motion studies in an attempt to evidence the impact on the workplace. The quality of articles as measured by the QuADS tool^21^ was variable, which may be influenced by the wide scope of the review research question, or it may demonstrate potential evidence gaps in this field. However, the varying quality of articles may be due to the interpretation of some of the criteria used within the tool to measure quality. A criterion that most articles scored poorly on was “justification for analytic method selected”; most articles did not provide justification, but this does not necessarily mean that the method of analysis was inappropriate. Indeed, most articles scored highly for the criterion of “the method of analysis was appropriate to answer the research aim/s”, suggesting that the quality of the research itself was good, however the articles did not always include the rationale for their analysis strategy. Another criterion that generated low scores for a large proportion of articles in this review was “Evidence that the research stakeholders have been considered in research design or conduct”. This may be due to the wide spread of time the articles cover, as stakeholder involvement in research is a more recent expectation within health care research.^61^

### Deployment, Scope of Practice, and Role Differentiation

This review suggests that the deployment of SWAPs is varied, and often dependent on setting, eg, rural versus metropolitan locations,^44^ and allied health discipline, eg, podiatry versus occupational therapy.^55^ Variation also occurs within individual allied health disciplines,^31^,^52^ indicating a lack of clear profession-based models of deployment for SWAPs, or the adoption of transdisciplinary models.^62^,^63^ This variation is exacerbated by widely differing services and patient populations. Other elements of the utilization of this workforce are inconsistent, such as supervision, team structure, and entry route into the vocation. This highlights the lack of professional workforce models for the deployment of this workforce over the last few decades. Acknowledging that new frameworks may take time to become embedded in workforce practices, variation in SWAP workforce deployment is expected in response to the struggles that health care services are experiencing in recent years, with some services being under more pressure than others. For example, one Australian study^55^ showed that rural and remote areas had higher proportions of SWAPs than metropolitan areas, and as rural and remote areas in Australia have been associated with poorer health outcomes,^64^ this is likely to increase the demand on health services in these areas. This could suggest that higher proportions of SWAPs have been employed as a way of addressing this increased demand. However, this scoping review found no evidence of healthcare services making informed decisions on their proportions of SWAPs based on demand data.

Scope of practice also varies, with some articles showing the range of restrictions placed on the SWAP workforce, some demonstrating underutilization of SWAPs, and others describing examples of innovative practice. Scope of practice also varies depending on variables such as the supervising registered practitioner, the needs of the service, and team structure. Arguably this variation shows the complexity of ensuring that SWAPs are able to work to their full scope of practice without their duties creeping into the registered practitioner role. Indeed, two articles^34^,^58^ showed considerable overlap in the duties of SWAPs and registered practitioners, and another^38^ showed that certain settings were more likely to see SWAPs taking on the role of junior registered practitioner. This suggests that role creep is occurring in allied health settings, which may be due to a lack of understanding around the role boundaries of SWAPs.

### Differences in Setting and Discipline

Deployment differs depending on variables such as geographical setting and type of service, and level of delegation depending on AHP discipline. These findings may reflect the idea that different settings produce different ways of working. For example, one study showed that SWAPs working in outpatients were not allocated their own caseload due to the brevity of treatment in these settings, when compared with those working in other healthcare settings. Another study showed that delegation of patient-related tasks to SWAPs was more likely in exercise physiology and dietetics than those in physiotherapy, psychology, and podiatry, and also more likely in acute hospital settings than in community settings. It is not known why this is the case; however, the differences in deployment and delegation may simply reflect the needs of the service, suggesting that SWAPs can be a versatile and adaptable workforce.

### Factors Influencing Effective Deployment

The registered AHP workforce can be a barrier to effective deployment of SWAPs. Reluctance of AHPs to delegate was frequently cited as a factor that impeded the effective utilization of this workforce, though this differed depending on setting and profession, and level of trust between the registered professional and SWAP. Delegation appears to increase over time, suggesting that once trust had been developed and competence demonstrated, AHPs were more comfortable with delegating more highly skilled work to SWAPs. The concept of earning AHPs’ trust before being able to work to their full scope of practice may be linked with a lack of consistency in training and education for SWAPs. Indeed, numerous entry routes into SWAP roles were noted, which may result in poor understanding of how SWAPs are trained and educated, and the skills and competence that they achieve through these routes. This review identified that registered practitioners have reported feeling threatened by role extension,^26^,^47^ and that managers have perceived registered staff to experience a reduction in job satisfaction due to the loss of delegated work.^26^ These factors may also contribute to AHPs’ reluctance to delegate to SWAPs. Interestingly, an article in this review identified that registered practitioners felt unprepared to provide supervision for SWAPs,^29^ which may suggest inadequate pre-registration training and a potential for inconsistent and unsatisfactory supervision. A previous systematic review^16^ also found that registered practitioners were not provided with training in delegation to or supervision of SWAPs and described a lack of clarity over what tasks can be delegated. This added pressure on registered practitioners, already overburdened with increased demand on their service, is unlikely to foster healthy and positive working relationships between registered practitioners and SWAPs. However, a recent article^52^ highlighted positive relationships between radiographers and SWAPs, although this finding was based on SWAPs’ willingness to seek support and assistance from registered practitioners. This could suggest that SWAPs seeking advice from registered practitioners enables a constructive rapport to be formed.

### Career Pathway

This review identified that despite there being vision and motivation for the SWAP role to develop, barriers are impeding this, such as the absence of a defined career framework, pathway and training plan, and limited opportunities for training or experience. One recent article^41^ included within the review proposed a career pathway to optimize the utilization of SWAPs, which suggests progression and steps towards defining the role. However, two potentially influential frameworks have been published recently, an Australian “Allied Health Assistant Framework”^65^ in 2020, and a UK “AHP Support Worker Competency, Education, and Career Development Framework”^12^ in 2021. The UK framework aims to standardize grading, education, training, and scope of practice within the National Health Service (NHS) in England. The production of these frameworks demonstrates an increasing policy, political and professional commitment to defining and developing SWAP roles. Several articles in this review also provided subtle evidence that role progression and relaxing of supervisory requirements is occurring.^22^,^33^,^35^,^52^ Whilst this progression is promising, frustration with the limits to the scope of practice is still reported, as are limited career opportunities.^30^,^32^ These national frameworks need to address these and other barriers to enable effective deployment of the SWAP workforce. This could be achieved through the development of profession-specific frameworks. The current SWAP frameworks are multi-professional which is likely to be too reductive when considering the variety of professions and disciplines covered within the AHPs. Discipline-specific frameworks could provide clarification on factors such as the level of supervision required for SWAPs, scope of practice, required training and education, and setting-dependent adaptations. Clearly defining these elements may also provide reassurance to AHPs who are not confident in delegating to SWAPs. A previous systematic review^15^ identified a lack of clarity on role boundaries between the different levels of SWAPs; discipline-specific frameworks could provide clarity on this area and improve the career pathway for SWAPs by clearly defining the necessary steps to move through the pathway.

### Impact on Service

Results from this review highlight that the utilization of SWAPs helps to improve service delivery, and anecdotal evidence suggests that they enable a cost-effective way of providing a health and care service. This corresponds with the findings from a 2010 systematic review^14^ that showed that SWAPs improved patient outcomes. However, none of the included research studies for this scoping review conducted any formal analysis of the cost-effectiveness of models of deployment for this workforce, a finding also identified in a 2013 systematic review^15^ of the effectiveness of advanced SWAPs, and indeed in a 2021 systematic review^16^ of cost effectiveness of delegation to AHP support staff. As the lack of evaluation of cost-effectiveness of SWAPs is being reported repeatedly in systematic reviews over a considerable timeframe, this suggests that this avenue of inquiry is not being prioritized in research at potential detriment to the recent attempts to progress the roles in the form of frameworks. While SWAPs may typically earn a lower salary than the registered practitioners they are working alongside, this does not necessarily mean that they therefore provide a more cost-efficient service. SWAPs may free up registered practitioners to see more complex patients, but if SWAPs are not as efficient with the less complex patients there is no overall cost benefit. Equally, if significant SWAP supervision is required by registered practitioners, this does not release registered practitioners to complete more complex, or additional work. An economic evaluation of changing the skill mix would allow for a true assessment of the cost-effectiveness of this workforce. Indeed, this would be extremely valuable in light of the financial difficulties healthcare providers are currently facing.

### Future Research

This review highlighted that research examining the deployment of SWAPs in AHP settings is limited. Many studies are of low quality, small in scale, and rely on gathering staff perceptions rather than conducting objective evaluations of the impact of SWAPs on service delivery, including cost-effectiveness. Large-scale, rigorous studies are required to fully understand the impact of this workforce and investigate its currently untapped potential. Additionally, future research should incorporate relevant SWAP frameworks, as this may help to reduce the variation seen in existing research.

## Limitations

The majority of the included articles originated from the UK or Australia and therefore the implications are not easily generalized to other international settings. Only articles published in the English language were sourced, which may have contributed to the lack of diversity in the country of origin. However, the lack of articles from other countries may suggest that workforce researchers are not currently focusing on this aspect of the health services workforce.

A further limitation of the current study is the screening process. While the database searches followed a robust process for screening articles, the search conducted in Google Scholar only employed one of the authors to complete the initial title and abstract screening. This may have introduced bias into the selection of articles imported; however, as this screening came after the initial stringent screening from the database literature search in which the solo coder took part, bias is likely to be minimal. Similarly, the search strings (Appendix 1) may have been too narrow when considering the varied titles used to describe SWAPs, which may have resulted in some articles being missed. Additionally, relevant articles may have been missed due to omission of hand-searching relevant journals and searching the bibliographies of included articles. While these steps are not mentioned in the scoping review guidance that was followed for this scoping review,^18^ they are mentioned in the Arksey and O’Malley scoping review guidance.^17^

A large proportion of articles included in this review used qualitative methods with the findings often based on individual perspectives. While helpful in providing rich, informative data, caution should be employed when interpreting the results of such studies as there may be other variables affecting these outcomes, such as social desirability bias.

## Conclusion

Many AHP health services have rising demand coupled with static or reducing financial resources. In many countries and professions, this is combined with a workforce crisis, with many departments carrying high levels of vacancies in their registered workforce. Effective deployment of SWAPs can not only plug the gaps in the service, but as shown by this review can also positively impact patient experience and outcomes. However, this review has identified highly variable deployment of SWAPs, and for this workforce to be utilized to its full potential work must be undertaken to ensure that role boundaries and career pathways are clearly defined for each individual AHP discipline. While the recently published AHP-wide frameworks will provide much-needed clarity, the needs of each AHP profession and the settings within which they work are likely to differ significantly. Professional bodies have a responsibility to guide their respective managers, practitioners, and SWAPs in the interpretation of the AHP frameworks within their professional setting, showcasing models of effective deployment, and supporting extensions of the scope of practice where patient care will ultimately benefit.

This review shows that SWAPs are a valuable but largely underutilized workforce that is prevented from reaching its full potential due to inconsistencies in deployment, a lack of clarity in role definition, variation in education and training, and the absence of a clearly defined career pathway. The limited progress that has been achieved is also disadvantaged by the “one size fits all” approach to current AHP-wide frameworks, with a need for more profession-specific guidance. Research investigating the impact and cost-effectiveness of different models of deployment of this workforce is limited and mostly qualitative or anecdotal. Qualitative research is valuable in this field as it enables different staff perspectives to be examined; however, further quantitative evaluation studies are needed to provide more objective evidence of the impact of SWAPs.
